# Supplementary material for: Vitamin D3 Repletion Improves Vascular Function, as Measured by Cardiorenal Biomarkers in a High-Risk African American Cohort
Source: Nutrients. 2022 Aug 14;14(16):3331. doi: 10.3390/nu14163331 (PMC9414215; doi:10.3390/nu14163331)

Figure S1. Distribution and log transformation of plasma full-length osteopontin (flOPN), fibroblast growth factor-23 (FGF-23), and plasminogen activator inhibitor-1 (PAI-1)

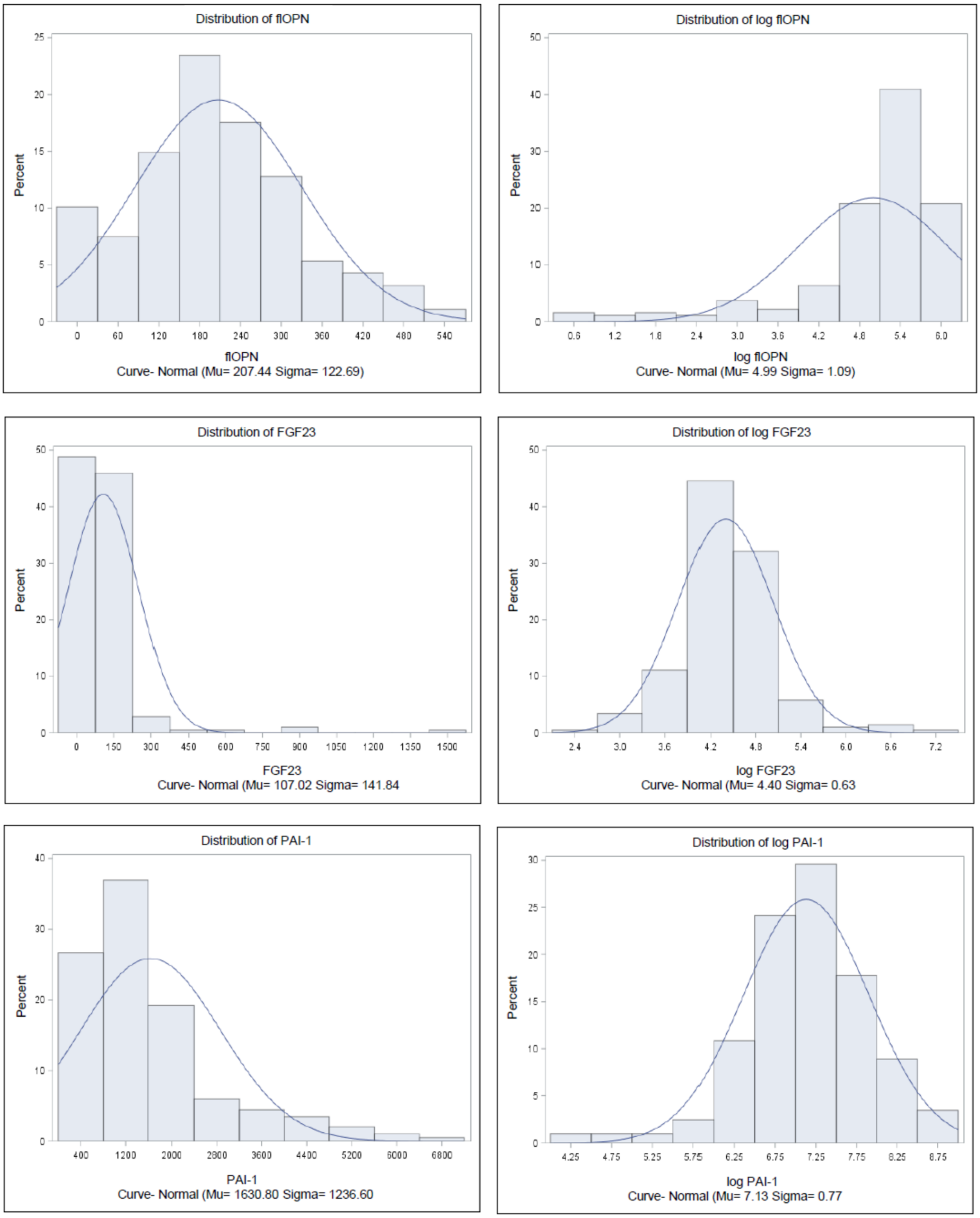

Supplement: Supplementary file 1 [file nutrients-14-03331-s001.zip › nutrients-1783498-supplementary.pdf]
